# Artificial Intelligence in the Management of Facial Palsy: A Narrative Review Across the Care Continuum

**DOI:** 10.1177/22925503261490552

**Published:** 2026-09-29

**Authors:** Adham Elsherbini, Jonah Perlmutter, Ibrahim Durowoju, Adam Mosa

**Affiliations:** 1 Faculty of Medicine, 12366 University of Toronto, Toronto, ON, Canada; 2 Division of Plastic and Reconstructive Surgery, 7979 The Hospital for Sick Children, Toronto, ON, Canada

**Keywords:** Artificial intelligence, machine learning, facial reanimation, apprentissage machine, intelligence artificielle, réanimation faciale

## Abstract

**Introduction:**

Facial paralysis produces substantial functional and psychosocial impairment which facial reanimation surgery seeks to mitigate by restoring facial movement and improving symmetry. However, despite technical advances in nerve- and muscle-based reconstruction, perioperative evaluation and outcome assessment remain largely subjective and variably reproducible. Artificial intelligence (AI) and computer vision have emerged as tools to provide objective, scalable assessment across the facial reanimation care pathway.

**Methods:**

MEDLINE, Embase, and the Cochrane Library were searched extensively to identify studies evaluating AI applications relevant to facial reanimation surgery. Included studies were screened by three independent reviewers and mapped to stages of perioperative care using an evidence-mapping framework.

**Results:**

Thirteen studies met inclusion criteria and were distributed across multiple stages of care. Evidence was most concentrated in diagnosis and severity assessment and in postoperative follow-up, where AI systems automated facial grading scales, quantified asymmetry, and extracted dynamic motion features from photographs and video. Several machine learning and deep learning models demonstrated high diagnostic accuracy and strong correlations with clinician-assigned scores, often exceeding inter-rater reliability of traditional grading systems. Fewer studies addressed prognostic modeling, preoperative planning, or rehabilitation, though emerging applications included outcome prediction, three-dimensional symmetry analysis, and AI-assisted evaluation of spontaneous emotional expression after reanimation.

**Conclusion:**

AI applications in facial paralysis care are most mature in assessment and outcome measurement, where they offer improved objectivity and reproducibility. However, broader clinical integration will require larger, diverse datasets, prospective validation, and alignment with ethical and regulatory standards to demonstrate meaningful patient centered benefit.

## Résumé

**Introduction:**

La paralysie faciale est responsable d’une incapacité fonctionnelle et psychosociale importante, que la chirurgie de réanimation faciale cherche à atténuer par le rétablissement du mouvement facial et l’amélioration de la symétrie. Toutefois, malgré les progrès techniques en reconstruction nerveuse et musculaire, l’évaluation périopératoire et celle des résultats demeurent largement subjectives, et leur reproductibilité est variable. L’intelligence artificielle (IA) et la vision par ordinateur permettent désormais une évaluation objective et adaptable tout au long de la trajectoire des soins en réanimation faciale.

**Méthodologie:**

Les chercheurs ont effectué une recherche approfondie dans Medline, Embase et la Bibliothèque Cochrane afin d’en extraire les études qui évaluent des applications de l’IA utiles en chirurgie de réanimation faciale. Trois réviseurs indépendants ont analysé les études retenues et les ont cartographiées en fonction des étapes des soins périopératoires selon un cadre de cartographie des données probantes.

**Résultats:**

Treize études, réparties entre de multiples étapes des soins, respectaient les critères d’inclusion. Les données probantes touchaient surtout le diagnostic, l’évaluation de la gravité et le suivi postopératoire, et les systèmes d’IA étaient surtout utilisés pour automatiser les échelles de classement facial, quantifier l’asymétrie et dégager les caractéristiques de mouvement dynamique à partir de photos et de vidéos. Plusieurs modèles d’apprentissage machine et d’apprentissage profond présentaient une précision diagnostique élevée et de fortes corrélations avec les scores attribués par les cliniciens, souvent supérieures à la fiabilité interévaluateur des systèmes de gradation traditionnels. Peu d’études portaient sur la modélisation pronostique, la planification préopératoire ou la réadaptation, mais les nouvelles applications incluaient la prédiction des résultats, l’analyse de symétrie tridimensionnelle, et l’évaluation assistée par IA des expressions émotionnelles spontanées après la réanimation.

**Conclusion:**

Les applications de l’IA les plus avancées pour la prise en charge de la paralysie faciale concernent l’évaluation et la mesure des résultats, car elles améliorent l’objectivité et la reproductibilité. Toutefois, pour en accroître l’intégration clinique, il faudra des ensembles de données plus vastes et plus diversifiés, une validation prospective et le respect des normes éthiques et réglementaires qui fourniront d’importants avantages démontrés centrés sur le patient.

## Introduction

Facial paralysis, characterized by unilateral weakness or paralysis of the face from cranial nerve VII dysfunction, results in a serious impact on patient quality of life through several domains. Functionally, patients have difficulty drinking, speaking, reduced eye protection, corneal dryness, or ulceration.^1^ Psychosocially, nonverbal communication and cosmetic appearance are impacted. One study of 920 patients with facial palsy showed that the severity of the palsy was correlated with 26% of the negative variance in the quality of life of patients.^2^

Facial reanimation surgery seeks to restore symmetry, dynamic movement, and emotional expression in patients with facial paralysis. Although modern nerve- and muscle-based procedures have advanced substantially, perioperative evaluation and outcome assessment remain limited by subjective grading scales, variable inter-rater reliability, static photography, and inconsistent reporting of dynamic facial function. These limitations are particularly important in reanimation surgery, where clinically meaningful success depends not only on resting symmetry but also on smile excursion, spontaneity, synkinesis, and patient-perceived social expression.

Artificial intelligence (AI) and computer vision are increasingly being applied to facial paralysis care, driven by the need for more objective, scalable, and reproducible assessment of facial function. A recent systematic review by Müller et al synthesized AI-based facial assessment tools used in facial palsy and proposed videographic standards and software-selection guidance.^3^ However, AI applications relevant to facial reanimation remain scattered across heterogeneous tasks, data modalities, model architectures, and outcome measures. This fragmentation limits understanding of where the evidence is strongest, where validation remains insufficient, and which stages of care have received little investigation.

To address this gap, this narrative review organizes AI applications across the facial reanimation care continuum, from baseline severity assessment and preoperative planning to postoperative outcome measurement and rehabilitation. By mapping studies according to stage of care and intended clinical use, we aim to clarify where evidence is most mature, where validation remains limited, and which clinically important areas remain underexplored.

## Methods

MEDLINE, Embase, and Cochrane were searched for articles discussing AI applications in facial reanimation. Search terms included “AI”, “machine learning (ML)”, “computer vision”, “facial reanimation”, “facial paralysis”, and “reanimation surgery”. Articles were included if they evaluated or discussed AI-based tools relevant to baseline assessment, prognostication, surgical planning, postoperative outcome measurement, or rehabilitation in facial palsy or facial reanimation. All articles were screened for eligibility by three independent authors (AE, JP, ID). Included studies were categorized according to their application across predefined stages of care; studies addressing more than one stage were mapped to all applicable categories.

## Result

Thirteen studies met inclusion criteria and were mapped to all applicable stages of care. Evidence was most concentrated in Diagnosis and Severity Assessment (38% of included studies) and Follow-Up and Outcome Measurement (62%), where AI was primarily used to automate grading, standardize clinician-assigned scoring, and quantify dynamic facial function from photographs or video. Other stages were less represented, including prognosis and predictive modeling (8%), preoperative planning and intraoperative support (15%), and rehabilitation and nonsurgical treatment (8%) (Table 1). Because studies could be mapped to more than one stage, proportions across stages were not mutually exclusive.

**Table 1.** Evidence map of AI Applications Across Stages of Facial Palsy and Facial Reanimation Care.

Continuum Stage	Density	Coverage (n/N, %)	Use Cases
Diagnosis and severity assessment	Moderate	5/13 (38%)	Automated grading / standardized scoring from photo/video/depth (incl. eFACE-like tools)
Prognosis and predictive modeling	Low	1/13 (8%)	Retrospective risk prediction (eg, synkinesis) using early clinical ± electrophysiology features
Preoperative planning and intraoperative support	Low	2/13 (15%)	Sparse planning comparisons ± largely conceptual intraop AI nerve monitoring/support
Treatment execution (rehabilitation and nonsurgical)	Low	1/13 (8%)	Early-stage assistive/closed-loop stimulation concepts (simulation/feasibility)
Follow-up and outcome measurement	High	8/13 (62%)	Objective postop quantification (symmetry, smile dynamics, AUs/emotion, spontaneous smile metrics)

### Diagnosis and Severity Assessment

An accurate quantification of facial paralysis is crucial for guiding management and evaluating postoperative recovery. Traditionally, clinicians’ grade facial nerve function using the House-Brackmann (HB) scale or Sunnybrook grading,^4^ both relying on subjective observation of facial symmetry and movement. Despite the HB scale providing a severity stratification, the model lacks granularity and inter-rater consistency,^4^ rendering computer vision and ML as potential avenues to address facial motion analysis. Gaber et al introduced a real-time Kinect V2 system for extracting facial animation units (FAU) to classify paralysis severity across seven grades. Using 375 patient and 1650 control records, they trained an ensemble of SVMs (stacking ± bagging with hybrid imbalance handling). Using five-fold cross-validation, the ML model yielded a 96.8% accuracy, 88.9% sensitivity, and 99% specificity, confirming the model's robustness and stability.^5^ They conclude that Kinect-derived FAUs can provide a detailed, objective facial paralysis grading system that outperforms traditional subjective scales. Alternatively, Rao et al developed a video-based ML framework that analyzes full-motion clips, using techniques such as optimal transport and Mahalanobis-distance analysis, to detect subtle reinnervation cues during movement. It then translates these continuous measurements into predicted HB and eFACE scores, proving significantly more sensitive than traditional exams or static photo analysis.^6^ Monini et al applied ten gray circular markers at standardized facial points on subjects with varying HB grades (I-V) during resting and ordered facial movements to computer inter-marker distance differences between sides to derive a global facial paralysis index. Preliminary results in 50 subjects showed clear differentiation of facial paralysis grades, with global index ranges (normal 6.2–7.9; HB II 10.6–18.9; HB III 22.2–33.1; HB IV 38.6–49.8; HB V 51.0–66.9).^7^

Another study, Feng et al, proposed MLSTNet, a novel three-stage multitask learning network that integrates static facial appearance and dynamic texture extraction with spatial-temporal disentanglement for video-based grading of facial paralysis severity.^8^ They first extract static heatmaps via a frozen pretrained face detection backbone and dynamic heatmaps via optical flow estimation, then fuse these proxy tasks through a lightweight encoder for binary classification of paralysis presence, and finally capture spatial and temporal dependencies with an attention-based disentanglement network for six-level severity grading.^8^ The model was trained and validated on a synthesized dataset of 1241 videos drawn from three independent datasets. MLSTNet achieved high accuracy (95.14%), precision (95.61%), recall (95.43%), and F1-score (95.26%).

With regard to deep learning (DL), Shi et al developed an improved Single Shot Detector network for graded assessment of Bell's palsy severity by replacing the VGG backbone with a more efficient feature extractor and integrating scientific computing modules.^9^ They trained the model on facial images of Bell's palsy patients, using DL-driven facial attribute recognition to automate the grading process. The network achieved an average precision of 87.9% and recall of 88.3% across the three severity levels, while significantly reducing computational burden.^9^

Notably, Zhang et al conducted a systematic review on AI-based facial palsy evaluation systems and reviewed public datasets and key studies since 2008. The authors highlight major challenges, including small and variable datasets with a lack of clinical validation. They conclude that, although AI holds promise for objective, reproducible grading, large-scale data collection and rigorous validation are needed before routine clinical use.^10^ However, beyond grading severity, AI applications can characterize complex movement disorders associated with facial paralysis. AI applications have the potential to track Bell's palsy synkinesis and calculate severity, guiding Botox treatments and monitoring change over time. Kishimoto-Urata et al applied ML algorithms to early clinical data from Bell's palsy patients to predict which patients would develop significant synkinesis months later.^11^ Using initial electroneurography results, the severity of paralysis, and an automated analysis of blink dynamics, the ML-based logistic regression model achieved an area under the receiver operating characteristic (AUC-ROC: ability to discriminate positive from negative cases) score of approximately 0.90 in predicting later synkinesis.^11^

### Preoperative Planning and Intraoperative Support

Traditionally, preoperative decision-making has been guided by the surgeon's expertise and case series published in the literature. However, significant variability in practice remains. By leveraging datasets of prior patients, AI aims to augment preoperative planning through outcome prediction and patient-specific anatomical mapping. Woo et al retrospectively compared one-stage dual innervation (CFNG ± masseteric) versus masseteric-only free gracilis transfers in 35 patients using an AI-based 3D symmetry analysis.^12^ At 1 year, the dual-innervation group showed significantly greater improvements in resting mouth-corner distance (2.55 ± 2.94 mm vs 0.52 ± 2.75 mm, p = 0.048) and angle symmetry (1.21° ± 1.43° vs 0.02° ± 0.22°, p = 0.001), as well as better smile angle symmetry (3.20° ± 2.38° vs 1.49° ± 2.22°, p = 0.041), demonstrating superior postoperative facial symmetry with dual innervation.^12^

Preoperative imaging can reveal the status of the facial nerve and surrounding muscles, however, interpretation of these modalities is time-consuming and varies with clinician expertise.^13,14^ AI can assist by processing imaging data to map out critical structures. In an editorial, Ali et al described experimental systems in which AI analyzed imaging to create a detailed 3D model of a patient's facial nerve, identifying safe and high-risk areas for surgery.^15^

### Follow-Up and Outcome Measurement

In the current paradigm, postoperative monitoring relies on subjective grading, including clinical observations or semiquantitative measurements of the nasolabial angle or ruler measurements on photographs.^16^ These assessments are prone to bias, enabling AI to provide more calculated outcome measurements. Boonipat et al evaluated the use of a commercially available AI-based facial expression recognition system (Noldus FaceReader) to quantify emotional signals before and after facial reanimation surgery.^17^ They retrospectively analyzed pre- and postoperative video clips of 15 patients who underwent CFNG with free gracilis muscle transfer and compared them to eight healthy controls, measuring the proportions of seven basic emotions during neutral repose and posed smiles.^17^ Postoperatively, the “happy” signal in posed smile videos increased significantly from 13% preoperatively to 42% postoperatively (p < 0.0001), approaching the 53% happy signal in control smiles.^17^ Concurrently, the sad signal decreased from 15% to 9% (p = 0.092) and the neutral signal from 57% to 37% (p = 0.0012).^17^ These findings provide the first proof-of-concept that ML can objectively quantify changes in emotional expression following facial reanimation surgery. Similarly, Kollar et al evaluated a novel AI-driven video analysis (FaceReader v8.1) as an objective tool for functional outcome measurement after reanimation surgery.^18^ In this retrospective cohort study, they analyzed pre- and postoperative video recordings of 63 patients and 12 healthy controls, computing intensity scores for emotions and facial action units.^18^ The AI-derived intensity scores correlated strongly with the clinician graded Terzis score (r = 0.80 for happiness, r = 0.74 for lip puller). The authors demonstrate that AI-driven video analysis can serve as a reliable, objective complement to traditional human scoring systems in smile reanimation.^18^ Another prevalent metric is the eFACE score, a 15-item graded assessment of different facial regions and functions which yields a composite score out of 100. Miller et al, compared clinician graded eFACE scores with Auto-eFACE, an automated ML-enhanced facial palsy assessment tool on a dataset of 160 photographs.^19^ Auto-eFACE detected subtle asymmetries in normal faces, producing significantly lower symmetry scores for normals (93.83 ± 4.37 vs 100.00 ± 1.58, p = 0.01).^19^ For patients with flaccid paralysis and severe synkinesis, Auto-eFACE yielded higher symmetry scores than eFACE (59.96 ± 5.80 vs 52.20 ± 3.39 and 62.35 ± 9.35 vs 54.22 ± 5.35, respectively; both p = 0.08).^19^ Furthermore, in Rao et al's study on video analysis, the ML's continuous measurements were translated back into eFACE and HB values to validate the clinical findings.^6^ Similarly, Dusseldorp et al developed an automated ML-based pipeline combining facial landmark recognition (Emotrics) and emotion detection (Affdex) to quantify spontaneous smiling in facial reanimation cases.^20^ Validated humorous video clips were used to elicit spontaneous smiles from nine healthy subjects and 39 patients who underwent free muscle transfer, defining an “emotionality quotient” as the differential between joy and negative emotion scores.^20^ Normal subjects exhibited a median emotionality quotient of +100/0, whereas CFNG-innervated transfers scored +82/0, masseteric nerve–only transfers scored 0/−48, and dual innervation scored +10/−24 (joy p = 0.006; negative emotion p = 0.034).^20^ These results indicate that CFNG innervation yields the highest degree of spontaneous joy among reanimation techniques.^20^

## Discussion

AI applications in facial reanimation remain concentrated in baseline severity grading and postoperative outcome assessment, where computer vision and machine-learning tools show high accuracy and strong concordance with clinician-assigned scores, often outperforming the inter-rater reliability of HB and eFACE. By quantifying smile excursion, emotional expression, and three-dimensional symmetry, these systems provide greater reproducibility and granularity than static or subjective assessment. However, evidence is limited by small, retrospective, single-center cohorts with narrow demographic representation, raising concerns about generalizability and bias. Higher-impact perioperative applications, including prognostication, surgical planning, intraoperative support, and rehabilitation, remain underdeveloped. Translation will require prospective multicenter validation, diverse data-sharing frameworks, patient-centered outcomes, and regulatory alignment. Until then, AI should be viewed as an adjunct, rather than a substitute, for expert clinical judgment.

In addition to the aforementioned applications, an even more novel application lies in the development of systems that bypass the surgical repair altogether and directly restore facial movement through electrical or mechanical means. Functional electrical stimulation (FES) involves placing electrodes around paralyzed facial muscles and delivering electrical currents that induce muscle contractions.^21^ AI control of an FES system could allow for smarter control of the system. A proof-of-concept study by Steiner et al used the vestigial movement of the patient's ear muscles as a control signal, as many facial paralysis patients develop unintended contractions of tiny auricular muscles when attempting to move the face.^22^ The system places EMG electrodes over the auricular muscles to receive input when the patient attempted to blink or smile. A convolutional recurrent neural network (CRNN) was then trained to recognize the pattern of EMG signals corresponding to each intended facial action. The CRNN would decode the input signal and activate the stimulation electrodes on the paralyzed facial muscles. In Steiner et al's trial of 17 patients with chronic facial palsy and synkinesis, the model was able to correctly classify and trigger the desired movement with a median macro F1-score (the unweighted mean of all the per-class F1 scores, which are the mean of precision and recall) of 0.60.^22^

### Ethical and Regulatory Considerations

AI's role in facial reanimation surgery brings forth a complex array of ethical, legal, and regulatory challenges. While many of these issues mirror those encountered in broader medical AI implementation, the unique implications of facial palsy demand particular attention. For one, AI systems rely heavily on large datasets of highly sensitive facial images, video recordings of expressions, and potentially electrophysiological signals.^23^ Since facial data is inherently biometric and uniquely identifiable, there remains a profound invasion of privacy if the data is breached.^24^ All deployments must comply with prevailing data-protection frameworks (GDPR in Europe, HIPAA in the United States, or the EU's AI Act), including anonymization or deidentification wherever feasible and formal governance of data sharing and retention policies.^25^ Additionally, the performance of AI models is tightly linked to the diversity of the training data. Underrepresentation of certain demographics or severities of palsy can lead to systematic inaccuracies in assessment or post-operative monitoring, unfairly disadvantaging certain patient groups. To mitigate bias, datasets must reflect the full spectrum of demographic and clinical presentations and incorporate ongoing bias detection and fairness adjustment strategies.^25^ Furthermore, DL models often operate as “black boxes” making it difficult for clinicians to understand the mechanism behind specific outputs. In a surgical context, opaque recommendations can undermine clinical judgment and risk patient safety.^26^ Explainable AI techniques, such as visualizing the facial landmarks driving a palsy score or generating counterfactual simulations of proposed muscle transfers, can illuminate model reasoning. Beyond retrospective performance metrics, prospective controlled studies are needed to present tangible benefits, such as improved postoperative symmetry following AI employment. Further, robust failsafe mechanisms and human override controls must be built into guard against malfunctions or misclassifications. Informed consent processes should explicitly describe how AI metrics will be used, and patients should retain autonomy in discussing whether and how these measures inform perioperative care. Currently, emerging regulatory frameworks, such as the EU's high-risk AI classification or the FDA's SaMD guidance, are defining requirements for transparency and for postmarket surveillance to detect model drift.^27^

## Conclusion

AI has begun to transform several phases of facial reanimation surgery, including facial paralysis diagnosis, preoperative planning, and postoperative care. Computer vision and ML frameworks could quantify paralysis severity with greater granularity and reproducibility than traditional scales, guiding nerve- and muscle-based interventions. Despite these advances, significant barriers remain, including the need for large, diverse, clinically validated datasets, robust strategies to mitigate bias, explainable models that support surgeon decision making, and regulatory pathways that safeguard patient privacy and ensure long-term performance. Future work must address these challenges through multicenter trials, standardized data-sharing frameworks, and development of real-time systems.
